# Cardiovascular dysautonomia and cognitive impairment in Parkinson's disease (Review)

**DOI:** 10.3892/mi.2024.194

**Published:** 2024-09-19

**Authors:** Ibrahim Khalil, Reem Sayad, Ahmed M. Kedwany, Hager Hamdy Sayed, Ana Letícia Fornari Caprara, Jamir Pitton Rissardo

**Affiliations:** 1 Faculty of Medicine, Alexandria University, Alexandria 5372066, Egypt; 2 Faculty of Medicine, Assiut University, Assiut 71515, Egypt; 3 Department of Nuclear Medicine, Assuit University, Assuit 71515, Egypt; 4 Department of Neurology, Cooper University Hospital, Camden, NJ 08103, USA

**Keywords:** orthostatic hypotension, supine hypertension, dysautonomia, cognitive impairment, dementia, mild cognitive impairment, PD, non-motor symptoms, cardiac MIBG

## Abstract

Cognitive impairment is a prevalent non-motor symptom of Parkinson's disease (PD), which can result in significant disability and distress for patients and caregivers. There is a marked variation in the timing, characteristics and rate at which cognitive decline occurs in patients with PD. This decline can vary from normal cognition to mild cognitive impairment and dementia. Cognitive impairment is associated with several pathophysiological mechanisms, including the accumulation of β-amyloid and tau in the brain, oxidative stress and neuroinflammation. Cardiovascular autonomic dysfunctions are commonly observed in patients with PD. These dysfunctions play a role in the progression of cognitive impairment, the incidents of falls and even in mortality. The majority of symptoms of dysautonomia arise from changes in the peripheral autonomic nervous system, including both the sympathetic and parasympathetic nervous systems. Cardiovascular changes, including orthostatic hypotension, supine hypertension and abnormal nocturnal blood pressure (BP), can occur in both the early and advanced stages of PD. These changes tend to increase as the disease advances. The present review aimed to describe the cognitive changes in the setting of cardiovascular dysautonomia and to discuss strategies through which these changes can be modified and managed. It is a multifactorial process usually involving decreased blood flow to the brain, resulting in the development of cerebral ischemic lesions, an increased presence of abnormal white matter signals in the brain, and a potential influence on the process of neurodegeneration in PD. Another possible explanation is this association being independent observations of PD progression. Patients with clinical symptoms of dysautonomia should undergo 24-h ambulatory BP monitoring, as they are frequently subtle and underdiagnosed.

## 1. Introduction

Parkinson's disease (PD) is the second most common neurodegenerative disorder following Alzheimer's disease (AD). It affects an estimated 7 to 10 million individuals worldwide, a number that is projected to double by the year 2030 due to the aging of the population. The prevalence varies, ranging from 41 individuals per 100,000 individuals in the fourth decade of life to >1,900 per 100,000 individuals among those aged ≥80 years. The incidence, or the rate of newly diagnosed cases, tends to increase with age, although it may stabilize in individuals >80 years of age (https://parkinsonsnewstoday.com/parkinsons-disease-statistics/).

PD presents a diverse range of motor and non-motor symptoms. The main non-motor symptoms are neuropsychiatric features, insomnia, excessive daytime sleepiness and autonomic dysfunction. Anxiety and depression are common neuropsychiatric symptoms in PD, occurring from the early pre-motor phase to the advanced stages of the disease. These symptoms vary in intensity depending on the motor state, with anxiety being particularly prominent during ‘off’ ‘periods. Anxiety and depression often coexist, and it is crucial to identify the specific anxious depressive phenotype to effectively manage both conditions (1). Cognitive decline and dementia are typically regarded as a part of late-stage PD or as a result of aging; as many as 83% of individuals with PD may experience a certain degree of cognitive dysfunction (2). PD dementia (PDD) is associated with a marked decrease in cortical cholinergic activity. This decrease helps to explain why certain patients may have a limited clinical response to cholinesterase inhibitors (3). Early-stage PD often includes mild cognitive impairment (MCI), which, due to subjective cognitive decline, can be overlooked in clinical practice. The primary characteristic of this cognitive syndrome is a decline in executive function (4). Early cognitive impairment is classified as a frontostriatal condition that relies on dopamine and can be treated by dopaminergic medications, particularly concerning executive function (5). The majority of individuals with PD experience disruptions in their sleep and wakefulness, and the frequency of these disruptions tends to increase as the disease progresses over time (6). These abnormalities manifest via diverse mechanisms. Daytime drowsiness and sudden episodes of falling asleep can be distinguished from sleep problems that occur during the night. Nocturnal sleep disorders encompass several conditions, such as insomnia, which can be caused by an illness or medication and involve disrupted sleep and frequent, lengthy periods of waking up. Other disorders include rapid eye movement behavior disorder (RBD), periodic limb movements, restless leg syndrome and akathisia (7).

**Figure 1:**
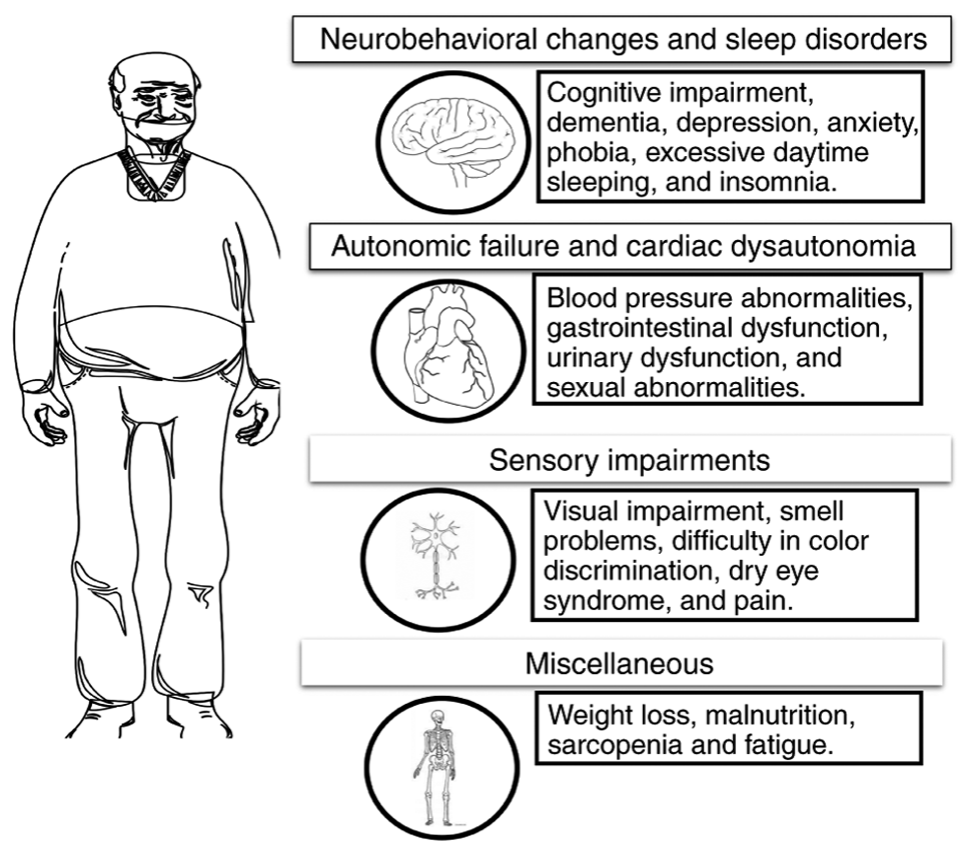
Summary of the non-motor symptoms of PD. The main non-motor symptoms are neurobehavioral features, sleep disorders, autonomic dysfunction and sensory impairments. Anxiety and depression are common neuropsychiatric symptoms in PD, occurring from the early pre-motor phase to the advanced stages of the disease. By contrast, cognitive decline and dementia are typically regarded as a part of late-stage PD. Autonomic dysfunction is a frequent occurrence in PD and can occur before the appearance of motor symptoms, and as the disease advances, the prevalence is increased. PD, Parkinson's disease.

Autonomic dysfunction is a frequent occurrence in PD and can occur prior to the appearance of motor symptoms. However, its prevalence increases as the disease advances. Autonomic issues involve failure in the bladder, bowel, and sexual functions, as well as cardiovascular complications, such as postural hypotension (8,9). Urinary dysfunction in PD encompasses symptoms, such as waking up at night to urinate (nocturia) and experiencing a higher frequency and urgency to urinate, which are linked to an overactive bladder muscle (detrusor hyperreflexia). The regulation of micturition relies on the autonomic arc of the sacral spinal cord segments. However, it is consistently facilitated by the pontine micturition center. The storage function, on the other hand, is facilitated by the hypothalamus, cerebellum, frontal cortex and basal ganglia (10-12). Bladder hyperreflexia in PD is considered to be associated with the absence of the inhibitory function of the basal ganglia. Imaging studies have revealed decreased dopaminergic function and an increased activity in the globus pallidus in individuals with PD with bladder dysfunction, as compared to patients with PD with normal bladder function (13,14). Gastrointestinal dysfunction is present across the entire gastrointestinal tract in PD, manifesting as excessive salivation, difficulty swallowing (dysphagia), delayed stomach emptying (impaired gastric emptying), constipation, and difficulty with bowel movements (impaired defecation) (15). The etiology of gastrointestinal dysfunction remains poorly comprehended and may encompass both external and intrinsic clinical and physiological alterations. Various neurotransmitters and neuromodulators regulate bowel function, such as acetylcholine, 5-HT, dopamine, noradrenaline, vasoactive intestinal peptide and nitric oxide (16). The cardiovascular system is significantly affected in PD, with various autonomic dysfunctions playing a crucial role. The heart is controlled by autonomic fibers that provide innervation, specifically sympathetic (noradrenergic and adrenergic) and parasympathetic (cholinergic) fibers. These fibers regulate both the heart rate and the force of its contractions. Up to 80% of individuals diagnosed with PD may exhibit cardiac autonomic dysfunction (17). This condition includes orthostatic hypotension (OH) and labile hypertension. OH is a sign of impaired sympathetic function and is frequently observed in PD, with a reported occurrence rate of 30-58% (18). Research has demonstrated that individuals with supine hypertension (SH) in PD are more likely to have end-organ damage, as well as an elevated risk of stroke and cardiovascular events (19). Sensory abnormalities are commonly reported, and a significant number of patients suffer pain throughout the progression of their condition (20,21). Various factors have been identified as the causes of pain, although a primary ‘central’ pain is also well-known (22). Prior clinical research has established that the basal ganglia plays a crucial role in sensorimotor functioning and action selection, both of which necessitate the integration of multisensory information (23). Postural instability is commonly attributed to disrupted motor programming in the basal ganglia. The basal ganglia directly affect the automatic control of postural muscle tone and postural reflexes by connecting them with the brainstem (24). A summary of the main non-motor symptoms is presented Fig. 1.

PD is a complex, progressive neurodegenerative disorder, and the presentation of the disease in each patient is unique (25). The severe depletion of dopaminergic neurons of the nigrostriatal system characterizes and likely produces the characteristic movement disorders (resting tremor, bradykinesia, rigidity and postural instability) in PD (26). However, the pathophysiology of PD involves dysfunction, not only in the dopaminergic system, but also in the cholinergic, serotonergic and noradrenergic systems. Therefore, as PD progresses, common non-motor symptoms, such as cognitive decline, sleep disturbances, mood disorders and gastrointestinal, genitourinary, or cardiovascular issues become more pronounced (27).

The origin of blood pressure (BP) abnormalities in patients with PD is multifactorial. It involves neurogenic factors related to PD pathophysiology, and both peripheral and central denervation. Additionally, treatment plays a role, as almost all dopaminergic medications (such as levodopa and dopamine agonists) can lead to decreased BP (28). Cognitive deficits manifest as impairments in executive functions, including planning, working memory and visuospatial attention. Over time, these deficits may evolve into clinically significant dementia. Even MCI has been identified as a predictor of declining functional outcomes (29).

Emerging evidence indicates a potential association between cardiovascular autonomic dysfunction and cognitive impairment in PD (30). Specifically, OH is associated with PDD (18). OH results from a dysfunction in the sympathetic noradrenergic system and is clinically significant in 20-50% of patients with PD (31). The present narrative review aimed to provide an overview of the current understanding of the connection between cardiovascular dysautonomia (focusing on OH, SH and non-dipping effect) and cognitive function in PD.

## 2. Spectrum of cardiovascular dysautonomia in PD

### Evaluation of cardiovascular dysautonomia. Different patterns of BP dysregulation associated with PD

OH can significantly affect individuals with PD, particularly during the later stages of the disease. OH is characterized by a decrease in systolic BP of at least 20 mmHg or a reduction in diastolic BP of at least 10 mmHg when standing or tilting the head up to at least 60˚ within a period of 3 min (32). Patients with recumbent hypertension should exhibit a decrease in systolic BP of at least 30 mmHg (33). The symptoms of OH, such as frequent fainting, lightheadedness, fatigue, nausea, trembling, headache, or pain in the neck and shoulder area, known as ‘coat-hanger pain’, may become more severe in the morning, after physical activity in hot environments, in the setting of dehydration, or after consuming alcohol. Symptomatic OH can cause significant disability and an increased risk of dangerous falls (34). In a previous study, syncope, which is a temporary loss of consciousness, was found in 4% of patients with PD and in 1% of the control group (35). The frequency of these symptoms increases as the disease advances (36). Screening for neurogenic OH (nOH) using orthostatic symptom questionnaires, orthostatic BP measurements and specialized autonomic testing may be beneficial for the identification of symptomatic and asymptomatic cases as cardiac sympathetic denervation and nOH can occur even during the early (premotor) stages of PD (37). One of the scales most frequently used in research for grading OH in patients with PD is the orthostatic grading scale (38).

Patients diagnosed with OH may also have SH, which is characterized by a systolic BP ≥150 mmHg or a diastolic BP ≥90 mmHg (39). SH typically does not cause any noticeable symptoms. However, some individuals may experience a throbbing headache when lying flat. It has the potential to cause ventricular hypertrophy (40), renal impairment (41) and intracranial hemorrhage (42).

As per the American Heart Association Council on High BP Research, the European Society of Hypertension, and the Japanese Society of Hypertension, nocturnal BP is considered normal if the average nighttime values are <120/70 mmHg. However, values >125/75 mmHg are considered abnormal (43-45). In a previous study, the nocturnal BP of patients who experienced a lack of sleep of at least 2 h lost its predictive value following a minimum of 7 years of monitoring for mortality and overall cardiovascular risk (46). However, in another study, the association between nocturnal BP levels and cardiac hypertrophy appeared less prominent when sleep was disrupted during overnight BP monitoring (47). Assembly data indicate that the measurement of nocturnal BP is a more accurate predictor of cardiovascular disease outcomes than daytime or 24-h BP measurements. As a result, nocturnal BP measurement is gaining significance in clinical practice (48-51). The clinical implications of nocturnal BP vs. daytime BP are more pronounced in treated hypertensive patients, as demonstrated by previous a meta-analysis of the International Database on Ambulatory BP Concerning Cardiovascular Outcome. This analysis included 7,458 participants with a mean age of 57 years. The findings indicated that daytime BP was a significant predictor of cardiovascular events in treated hypertensive patients after adjusting for nighttime BP (52). Nocturnal BP dipping refers to the normal reduction in nocturnal BP compared to daytime BP. While it may seem random, a 10-20% reduction in nighttime BP compared to daytime BP is generally considered normal. The term ‘non-dippers’ is used to refer to a specific group of patients with hypertension who experience a nocturnal BP decline <10/5 mmHg and have a high risk of stroke (53). Individuals classified as non-dippers have a higher prevalence of heart hypertrophy, silent cerebral infarction and microalbuminuria compared to those classified as dippers (54-56).

Postprandial hypotension (PPH) is characterized by a decrease in systolic BP ≥20 mmHg within a time frame of 2 h after consuming a meal (57). Food ingestion does not typically affect systemic BP in healthy individuals. However, alterations in gastrointestinal and pancreatic hormones trigger compensatory reactions in the heart and regional blood flow. Prior research conducted on individuals with autonomic failure has demonstrated that PPH can occur even when lying down due to the defective compensatory mechanisms for postprandial splanchnic blood pooling. Meals rich in carbohydrates are more likely to reduce BP than meals rich in protein or fat. PPH exacerbates OH (58). Studies have provided conflicting results as regards the degree of PPH in PD. Untreated PD has been shown to be associated with a slight decrease in BP after eating while lying down (59). It has been observed that older patients with PD experience a higher occurrence and severity of PPH compared to OH (60).

Post-exercise hypotension (PEH) is the temporary decrease in BP following a single exercise session. There are no specific standards for determining the extent and duration of the decline in BP after exercise that can be used to diagnose PEH (61). Patients with autonomic failure experience a decrease in BP and a worsening of OH when exercising, even in a supine position (62). A consistent reduction in systolic and diastolic BP among runners immediately following a 4-h run at an approximate speed of 6 miles per hour (63). PEH symptoms, such as dizziness, blurred vision and syncope have been observed in individuals after engaging in several forms of physical activity, such as walking, jogging, cycling, swimming and resistance exercise (64,65). A further explanation of the definitions of the different patterns of BP in patients with PD is provided in Table I.

*Cardiovascular system autonomic tests assessment*. Various autonomic studies need to be employed to assess the type and extent of a cardiovascular autonomic lesion, as autonomic symptoms can vary greatly.

Heart rate is a basic indicator of autonomic function, and the study of its measurement has been performed for a long time in various traditions, even before the advent of modern medicine (66). Additionally, it is the sole indicator of autonomic function that can be readily and accurately assessed, making it well-suited for extensive population-based or epidemiological studies. A significant association has been found between an increase in resting heart rate over a period and an elevated risk of mortality from ischemic heart disease and mortality from all causes (67). Increased mortality is linked to a slower heart rate recovery following exercise (68). From a molecular standpoint, administering high doses of β-adrenergic blocking medications only results in a modest reduction (5-10 beats) in the resting heart rate. On the other hand, the blockage of muscarinic receptors with atropine leads to a significant rise (~40 beats) in the resting heart rate (69). These discoveries have led to the idea that the resting heart rate is mostly controlled by vagal tone. The loss of this tone can have pathological consequences, possibly as it usually helps to prevent life-threatening irregular heart rhythms (70).

Heart rate variability (HRV), similar to heart rate, can significantly correlate with morbidity and mortality (71). HRV assessed by time domain or frequency domain techniques, is a non-invasive approach to evaluate autonomic function by examining how neural mechanisms affect the sinus node (72). HRV in the time domain may exhibit normal patterns but decline in the mid and late stages of PD (73). Power spectral analysis of HRV can serve as a screening method to detect autonomic dysfunctions in patients with PD by identifying low resting LF and HF powers. PD can result in simultaneous impairment of both sympathetic and parasympathetic nerves (74). Besides heart rate and HRV, another key component in evaluating dysautonomia is the heart rate response to deep breathing (75).

Head-up tilting is a standard and valuable test for assessing cardiovascular autonomic function, particularly in detecting OH. This can also be accomplished in the clinic by transitioning from supine to seated or standing positions. Measuring BP continuously during the exam is preferable, but regular readings with an upper-arm sphygmomanometer can still be sufficient (76). In addition, several patients may be asymptomatic despite having OH, leading to a lack of recognition by doctors.

The BP reaction to the Valsalva maneuver (VM), where the pressure in the chest is increased to a maximum of 40 mmHg, relies on the functioning of the baroreflex pathways. This reaction is often abnormal in patients with autonomic failure and is commonly employed to assess cardiovascular autonomic function. Patients with PD with OH and up to 25% of patients with PD exhibit abnormal baroreflex-cardiovagal gain responses, which are determined during the VM (77). The baroreflex sensitivity (BRS) VM approach has exhibited a stronger association with cardiovascular autonomic function than the spontaneous BRS indexes derived by the sequence or spectrum method. BRS VM, as opposed to spontaneous BRS, has demonstrated a prognostic significance in predicting the presence of cardiovascular autonomic neuropathy according to the diagnostic criteria established by the composite autonomic scoring scale in patients with PD (78).

Ambulatory blood pressure monitoring (ABPM) is valuable for identifying SH and PPH or OH, which can significantly affect circadian BP patterns. PPH is most commonly observed following the initial two meals of the day (79). Non-dipping, as previously mentioned, is a lack of decrease in BP during the night, and it is frequently observed in PD and autonomic dysfunction, resulting in a reversal of the usual circadian BP pattern. Furthermore, 24-h BP recordings offer clinicians valuable insight into the daily variations in BP, aiding in the development of a treatment plan (including the timing of medication administration and choice of medication) for conditions such as PPH, OH and nocturnal SH (80).

The meal challenge test is used to identify the presence of PPH. Typically, BP is monitored while the patient is lying down and during fasting, both before and up to 120 min after consuming a standardized meal. PPH is described similarly to OH, characterized by a reduction in systolic BP of 20 mmHg after a meal (58).

When assessing patients with PD, both while they are taking medication and when they are not, it has been shown that their BP and heart rate increase less during exercise compared to individuals without PD. This phenomenon appears to be directly linked to the disease itself and is not influenced by the drug used to treat PD (81). Exercise testing can detect exercise-induced or PEH in patients with autonomic failure (63).

The association between plasma catecholamines, particularly noradrenaline and outcomes in congestive heart failure is a prominent example of this interaction (82). The association between elevated noradrenaline levels and deteriorating outcomes in heart failure supports the effective (but first contentious) utilization of β-blocking medications in heart failure treatment (83). The primary constraint of plasma noradrenaline (and adrenaline) in assessing autonomic function is that it provides a momentary peek of activity unless multiple measurements are taken throughout time. In addition, values evaluated in the plasma indicate the overall equilibrium between neural release, neural reuptake and various clearance methods. For instance, under certain situations, such as severe hypoxia, there may be noticeable enhancements in sympathetic neuronal activation but only minimal increases in plasma noradrenaline (84). Patients diagnosed with PD who do not have OH have been observed to exhibit minimal alterations in their baseline supine resting and orthostatic plasma norepinephrine levels. During the initial phase of PD without OH, there may be a modest increase in plasma norepinephrine levels in certain people. On the other hand, individuals with PD who experience OH may exhibit lower-than-normal levels of BP both when standing and at rest, indicating dysfunction of the autonomic nervous system (39).

The skin vasomotor reflex is a term used to describe the reduced cutaneous blood flow in the palm or sole provoked by certain procedures, such as deep inspiration, mental stress, and isometric exercise. Patients with Lewy body disease had a weakened cutaneous vasomotor reflex (85). The discoveries align with Lewy body disease findings in the raphe nucleus, a region that significantly impacts the cutaneous vasomotor reflex. Additionally, these findings may indicate the involvement of postganglionic sympathetic pathways, which are commonly affected in individuals with Lewy body disorders (86). The skin vasomotor reflex, which may be evaluated with a Doppler flowmeter, frequently undergoes changes in Lewy body illnesses following various adrenergic stimuli (87).

### Cardiac 123I-metaiodobenzylguanidine (MIBG) and other imaging studies

Cardiac denervation occurs due to the loss of postganglionic sympathetic neurons. These neurons release catecholamine neurotransmitters, which bind to cardiac adrenergic receptors (primarily B1 receptors). Catecholamine reuptake is a normal function of postganglionic neurons. MIBG is an analog of norepinephrine that utilizes the reuptake transporter system to accumulate in postganglionic sympathetic neurons (88). Rissardo and Fornari Caprara (89) found that the early and delayed registration heart-to-mediastinum ratios (H/M ratio) for diagnosing PD were 1.70 and 1.51, respectively. In addition, the mean cutoff for the early and delayed phases was 1.89 and 1.86(89).

Research has demonstrated reduced myocardial MIBG uptake in patients with PD, indicating cardiac sympathetic dysfunction (90). Notably, this reduction is evident even in early-stage PD, suggesting that myocardial MIBG scans could be valuable for the early detection of PD. In this context, Kim *et al* (91) discovered that OH was closely associated with cardiac sympathetic denervation observed on cardiac MIBG in patients with early and mild PD. Furthermore, the decrease in MIBG uptake is not exclusive to PD patients with symptomatic OH or other dysautonomic symptoms (17,92).

Positron emission tomography (PET) scans using 6-[18F] fluoro-dopamine (18F-DA), another catecholamine analog, have also shown decreased uptake in patients with PD (93). Tyrosine hydroxylase can also be used to address the functionality of cardiac sympathetic postganglionic neurons (94). MIBG and PET (6F-DA) scans have shown that cardiac sympathetic denervation occurs independently of OH. This suggests that damage to cardiac nerves may precede damage to peripheral autonomic nerves. The latter's dysfunction, leading to inadequate vasoconstriction, is considered a significant factor in the development of OH (95).

### OH

Patients with autonomic dysfunction often encounter hemodynamic abnormalities, such as nOH and SH, in addition to genitourinary and gastrointestinal deficits. OH arises due to sympathetic noradrenergic dysfunction and is clinically significant in 20-50% of patients with PD (18,96,97).

nOH is defined as a decrease in systolic BP ≥20 mmHg or a decrease ≥10 mmHg in diastolic pressure within 3 min after transitioning from a supine to a standing position, without any connection to cardiogenic causes or hypovolemia, which are classified as non-nOH. It is advisable to extend testing in certain patients, as they may experience delayed OH. In up to 50% of cases, delayed OH can progress to classic OH after 10 years (33,98).

The decrease in BP during an orthostatic test is influenced by factors, such as the duration of rest before measuring supine BP, the method of assuming an upright position (active standing or passive tilting) and the time spent standing. In PD, OH occurs more frequently after tilting than during simple standing and is often characterized by delayed onset (99).

Symptomatic OH manifests as lightheadedness, presyncope, syncope, dizziness, visual disturbances, generalized weakness and fatigue. Symptomatic and asymptomatic OH are both linked to a higher incidence of hospital admissions, falls and a reduced quality of life (100,101). The findings presented in the study by Longardner *et al* (102) support previous evidence on the strong association between OH and cognitive impairment in PD (31,103).

Various pathological mechanisms contribute to the association between OH and cognition. These mechanisms include cerebral hypoperfusion resulting from recurrent episodic hypotension, widespread neurodegeneration, and dysfunction in central and peripheral noradrenergic systems (103,104). OH is considered one of the predictors of PDD in addition to age, male sex and MCI (105).

In the study by Cicero *et al* (106), the association between cardiovascular autonomic unction and MCI was investigated in 185 patients with PD from two movement disorders centers. Among these patients, 52 had OH, which was notably more prevalent in those with a longer disease duration and was associated with amnestic MCI (106).

Some authors have investigated the association between OH and posture-related cognitive impairment in PD, comparing cognitive function between patients with PD with and without OH to healthy controls (107,108). While in the supine position, both PD groups exhibited cognitive deficits related to frontostriatal and visuospatial functions. However, a transient improvement in cognition upon assuming an upright-tilted position was observed only in the PD group with OH (PD-OH). Furthermore, among patients with PDD, there was a greater decrease in systolic BP and a more pronounced attention impairment during standing (109).

The study by Longardner *et al* (102) investigated cognitive impairment differences between symptomatic and asymptomatic patients with PD-OH. The PD-OH group exhibited lower Montreal Cognitive Assessment (MoCA) scores and experienced a more pronounced decline during the follow-up period. Of note, the presence of OH symptoms did not significantly affect cognitive deterioration (102).

The causative or associative nature of the association between OH and cognitive dysfunction remains uncertain (103). This uncertainty arises partly as certain studies lack adjustments for critical variables, such as comorbidities, medications and age. Further research is required to elucidate this complex association.

### SH

nOH is closely linked to SH, which represents a hemodynamically contrasting form of BP dysregulation. Approximately half of the patients with PD-OH have concurrent OH and SH (110,111).

While expert consensus remains elusive regarding the diagnostic criteria for SH, it is generally defined as having a systolic BP ≥140 mmHg (or 150 mmHg) or a diastolic pressure ≥90 mmHg following a minimum of 5 min of rest while in a supine position (112).

In addition, patients with SH frequently exhibit an abnormal nocturnal BP profile (113). SH may be associated with either a reduction in the typical nocturnal BP decline (usually ≥10%), referred to as ‘non-dipping’, or an elevation in nighttime BP, known as ‘nocturnal hypertension’ or ‘reverse dipping’ (114,115).

The underlying mechanisms of SH may arise from a combination of baroreflex failure and vascular hypersensitivity. SH is likely linked to less severe peripheral sympathetic denervation than OH alone. Independent risk factors for SH include an older age, the akinetic-rigid motor subtype (which predisposes to cognitive decline), and pre-existing hypertension (116,117).

Kim *et al* (113) examined a cohort of 87 patients; of these patients, 25 had normal cognition, 48 had MCI and 14 were diagnosed with dementia based on comprehensive neuropsychological tests. All the patients with SH demonstrated a certain degree of cognitive dysfunction (P<0.001 based on the sign test). In the majority of neuropsychological domains, the SH group exhibited more severe impairments compared with the group without SH. Notably, 78.6% of patients with dementia had SH, while none of the patients with normal cognitive function had SH (χ² 27.360, P<0.001). Additionally, mean cognitive hypertension impact profile (CHIPS) scores were higher in patients with SH than in those without SH (113).

Palma *et al* (118), following a complete evaluation of 57 patients (35 with probable multiple system atrophy, 14 with PD and 8 patients with pure autonomic failure), concluded that SH in patients with nOH was associated with an increased risk of target organ damage, cardiovascular events and premature mortality.

### Abnormal nocturnal BP

In PD, an irregular nocturnal BP profile often indicates autonomic dysfunction. Normally, BP follows a circadian rhythm, with a decrease >10% at night, known as ‘dipping’. The majority of patients exhibit either non-dipping patterns or reverse dipping patterns, known as ‘risers’. Non-dippers experience a loss of nocturnal BP decline, while reverse dippers exhibit increased BP values during the night (114,119).

In PD, disruptions in the circadian BP pattern have been linked to coronary heart disease, stroke and higher mortality rates. Additionally, these disturbances are associated with target organ damage, cognitive impairment in older adults, and an increased burden of white matter hyperintensities (WMHs) (120).

To the best of our knowledge, there are limited studies available in the literature regarding nocturnal BP and circadian rhythm alterations in patients with PD. Oh *et al* (120) concluded that in patients with PD, the presence of nocturnal hypertension was associated with an elevated WMH score. Nighttime systolic pressure is closely associated with white matter changes. Additionally, blunted HRV and a lack of nocturnal decline in heart rate are related to increasing WMHs scores. Of note, the non-dipping phenomenon does not appear to influence WMHs. These findings suggest that white matter alterations are linked to circadian autonomic dysfunction, particularly nocturnal hypertension, in patients with PD.

Moreover, the presence of WMHs is significantly associated with the risk of PDD (121). Consequently, circadian BP disruptions may contribute to cognitive impairment in PD through various distinct and independent mechanisms.

### Comparison between cardiovascular dysautonomia and cognitive impairment between PD and dementia with Lewy bodies

While cognitive impairment in PD may be linked to cardiovascular dysautonomia, including BP dysregulation, the association between these factors in dementia with Lewy bodies (DLB) remains uncertain. Oka *et al* (122) aimed to investigate whether cardiovascular dysautonomia affects cognitive function in Lewy body disease. Their study evaluated 99 patients with *de novo* PD (n=75) and DLB (n=24) using the mini-mental state examination (MMSE) and frontal assessment battery (FAB). Additionally, they estimated cardiac MIBG scintigraphy, assessed OH, SH and PPH, analyzed nocturnal BP changes in 24-h ABPM and evaluated constipation (122). The results of their study were that in DLB, cardiac MIBG uptake was reduced, and OH, PPH and SH were severely disturbed compared to PD. Additionally, the nocturnal BP decline in 24-h ABPM was lower in DLB. In PD, the failure of nocturnal decline was associated with MMSE scores after adjusting for other clinical features. Furthermore, the FAB was significantly associated with nocturnal BP decline, age, and SH in PD, but no significant associations among these factors were found for DLB (122).

The notable association between disrupted nocturnal BP regulation and cognitive or executive decline in PD may be attributed to impaired microvascular circulation or the infiltration of α-synuclein in the central nervous system. Conversely, the absence of an association between BP insufficiency and cognitive impairment in DLB implies that Lewy body pathology initially affects the neocortex, irrespective of autonomic nervous system involvement (122). A summary of the key differences between PD and DLB is presented in Table II.

## 3. Cognitive impairment

The cognition defect may occur before, after, or even at the diagnosis time of PD in a variable degree of severity (123). Previous studies have proven that patients with PD have a much higher risk of developing dementia than individuals without PD (124,125). The risk of developing dementia among these patients reaches ~24-31% (126). Following the diagnosis of PD by 10 years, approximately half the number of patients have reported a risk for dementia and, in most cases, they developed dementia after almost 20 years of diagnosis. Several risk factors can lead to dementia in these patients. It was noted that the risk of cognitive affection in PDD and AD is almost the same (3). These patients require assistance and may become dependent on others (127).

Recently, there has been increasing interest in the stages that precede dementia in cognitive impairment, particularly MCI (128). Studies have proven that almost one-quarter of patients who have parkinsonism only without dementia suffer from MCI (129). Other studies have proven that almost 20% of patients suffered from MCI at the time of diagnosis and this increased to ~50% in the fifth year of the disease (130-132). MCI is a transitional cognitive stage between normal and dementia (133). The course of MCI is very variable and may return to normal cognition in nearly one-quarter of patients (133). However, even after returning to normal, the case may deteriorate to dementia again (134).

Studies have proven that patients with PD who have memory issues at diagnosis and before having any treatment are at a higher risk for developing MCI than those who do not initially have memory issues (135,136). However, a number of factors can affect the deterioration of MCI, particularly the affective symptoms (137).

### Risk factors for cognitive impairment in PD

Males are more predominantly affected by cognitive dysfunction than females. A previous cohort study demonstrated that the majority of cognitive parameters, such as semantic fluency, MoCA and phenomic parameters, apart from MMSE, are worse in males which has exhibited no difference in both sex groups. In addition, OH and RBD mostly affect males (138). Augustine *et al* (139) noted no difference in the age of disease onset, motor symptoms, or diagnosis in each sex. However, measures on symbol Digit and Scales for Outcomes of Parkinson's Disease-Cognition (SCOPA-COG) were more improved in females (139).

On the other hand, Gao *et al* (140) noted lower MoCA scores among females with equal scores on MMSE in both sex groups. However, the lower educational level among females in their study may have caused this skewing result. Males record higher results in visuospatial abilities but lower results in verbal memory studies than females (141). This occurs similarly in the elderly and among patients with AD (142).

The association between cognitive function and male sex remains unclear. OH and rapid eye movement (REM) have been shown to be associated with lower cognitive function, and these symptoms are more common in males. However, all these studies (139,140,141) included affected patients in the early stages of the disease; thus, further research is required to determine whether this effect will last in the late stages of the disease (143). Postmortem studies have revealed that in PD, both cortical and limbic Lewy bodies are associated with a higher risk of dementia. Higher levels of α-synuclein, along with higher oligomeric forms, are associated with greater cognitive impairment (144-146). In addition, tau pathology and amyloid play a role in cognitive dysfunction (147,148). Smith *et al* (149) recently reported that a large number of patients with PD who have dementia had the same pathological findings of AD in the form of moderate to severe pathology (tau and amyloid-β). Moreover, the cerebrospinal fluid (CSF) of subjects with PDD has been found to be associated with less amyloid-β than affected patients without dementia or normal population. Low levels of amyloid-β lead to the progression of dementia (150,151).

Tau pathology, β-amyloid and α-synuclein strongly affect PD cognition (152). Lower α-synuclein levels exert a more potent effect on tau pathology than β-amyloid on cognition (149). When PD is associated with AD, cerebral amyloid angiopathy (CAA) is more evident among those having dementia than those without dementia (153). In addition, there is a strong connection between dementia and CAA with Lewy pathology (154).

PD usually leads to sleep disorders, which may affect cognitive function. Even in the early stages of PD with REM disorders, patients are at a high risk of developing MCI. The risk of developing MCI is higher among patients with REM at the baseline (155). In addition, during the early stages of the disease, mood changes may lead to further cognitive dysfunction. However, the association between depression and cognitive dysfunction remains unclear (156). Some studies have demonstrated a strong connection between mood changes and cognition, although others have not found such a connection (157).

Diseases such as hypercholesterolemia, hypertension, obesity and diabetes are associated with dementia and cognitive disorders (158). Furthermore, in PD, these factors may worsen cognition. It has been found that body mass index, hypertension and diabetes mellitus were associated with hyperintensity of the brain white matter, which indicates ischemia and predicts cognitive affection (159-161). A previous cohort study revealed that high levels of C-reactive protein, glycosylated hemoglobin and high levels of fasting blood glucose during the early stages of PD are associated with low scores on MMSE (162). Another study proved that a high body mass index at the baseline led to a more rapid decrease in cognitive function in the early stages of PD (156). On the other hand, patients who lose weight during the disease course can exhibit a more rapid decline in cognitive function (163,164). However, obese patients have a slower rate of cognition and affection, particularly in memory and language (164). Thus, the association between body weight and cognition affection appears to be very complex.

Changes in blood in the form of OH or SH that occur in PD may increase the risk of developing dementia (165). In the case of OH, the repeated hypoperfusion changes and changes in the cerebral blood flow may cause cognitive affection (166). SH can increase the risk of cerebral ischemia and, thus, cognitive dysfunction, as aforementioned (159). Other research has reported a minor effect of ischemic cerebrovascular lesions on PDD, to no relation between the ischemic lesions in the heart and dementia (167). Diabetes mellitus is also associated with a high risk of cognitive affection in PD. Studies have found shared pathways between PD and diabetes mellitus (168-171). Bosco *et al* (172) reported that insulin resistance and abnormal metabolism of glucose were more common among patients with PDD than among those who did not have dementia.

Urate is a natural and crucial antioxidant in humans and has an inverse effect on PD (173-175). The uric acid levels in patients with PD are considered to be associated with oxidation, chelation, genetics and apoptosis (176). The uric acid level is low in the CSF of patients with PD, whether they have dementia or not (177). A low level of uric acid is a poor prognostic factor for memory affection, attention and global cognition (178-180).

Neuroinflammation inversely and strongly affects the cognitive function in PD. The activation of microglial cells leads to the release of cytokines, such as INF-γ, IL-1β, IL-6 and TNF-α, damaging the dopaminergic neurons (181). in addition, the activation of microglial cells decreases glucose metabolism in the frontal lobe and other regions of the brain in patients with PD affected by dementia (182). Mitochondrial disorders increase oxidative stress pathways and astrocyte and glial dysfunction, leading to abnormal inflammation (183-185). There is a decrease in the activity of mitochondrial complex 1 and low levels of mitochondrial DNA in the cortex of patients with PDD (186).

Traumatic injury to the brain is a key factor for disability. This disability may be due to inflammation, oxidative stress, neuron death, blood-brain barrier breakdown, or brain edema (187). PD is mostly associated with frequent trauma (188). Schiehser *et al* (189) reported a greater decline in cognitive function among patients with PD who had brain injury than who had not suffered any trauma. The cognitive affection was mainly in the areas responsible for memory and execution (189). However, further studies are required to fully elucidate this matter.

Some genes may affect the risk of developing PD and contribute to PDD. One of these genes is apolipoprotein E (APOE) (190). According to a previous prospective study (191), this gene inversely affects cognition. However, another study in the United Kingdom found no association between this protein and cognitive affection after almost 5 years (192). Thus, the effect of APOE on cognition among patients with PD remains elusive. Microtubule-associated protein tau plays a key role in microtubule stabilization and assembly. The H1/H1 genotype of this protein increases the risk of developing PD. The H1/H1 genotype leads to the progression of dementia, although this effect decreases along the disease course (193). This may support the notion that the effect of this genotype on cognition occurs only at the early stages of the disease (143).

Brain-derived neurotrophic factor (BDNF) is present in a high amounts in the cortex and subcortex. One of its key functions in the substantia nigra during development is to establish the dopaminergic neurons (194). One of its variants is the G196A (Val66Met) polymorphism (195). A previous study demonstrated that in PD, patients who are carriers of the BDNF Met gene are at a high risk of developing cognitive disorders (196). However, other studies have not found such a connection (197-199). Thus, the effect of BDNF on cognition in PD remains unclear.

### Pathophysiology of cognitive impairment in PD

In Parkinsonism, neuropathology briefly consists of the loss of dopamine neurons from the substantia nigra and the abnormal deposition of α-synuclein, leading to the formation of Lewy bodies. Firstly, this deposition occurs in the olfactory system, monoaminergic and cholinergic neurons of the brainstem, leading to synaptic affection (200). When the patient is affected by AD and PD, the pathology is almost the same as previously described (201), apart from deposition and synaptic affection that occur in the limbic system rather than the brainstem (201). The cognitive defect that occurs in PD is considered to be due to neurodegeneration that occurs in the brain rather than a functional defect (202).

When MCI is associated with PD, there is more degeneration in the dorsal striatum and the associative caudate nucleus. These patients exhibit some preservation of dopamine neurons in the brain (203). However, dopamine neurons are markedly lost in PDD, particularly in the temporal, parietal and frontal cortex (203). Normally, dopamine plays a crucial role in maintaining cognition by reinforcing memory, attention, cognitive effort and visuospatial functions (204,205).

Neurons that synthesize noradrenaline are present in the locus coeruleus, which also produces neuromelanin in humans (206). These neurons encourage arousal and waking, and play a critical role in cognition, particularly in long-term memory, attention, working and behavior (207). The noradrenergic fibers are arranged into two regions, the hippocampus and frontal cortex, and are crucial for cognition (207). An association has been noted between neuromelanin produced in the locus and MCI in PD cases (207). In addition, if patients have PD along with RBD, there is a similar and positive association between neuromelanin reduction and cognition and OH (208,209). Moreover, a deficiency in the transporter of noradrenaline in the brain in PD is associated with OH and low levels of cognition (209). If the patient has both PD and OH (which is due to the cutting of noradrenaline supply to the heart), the subject will suffer from cognitive dysfunction (102). This may be due to cerebral hypofunction resulting from OH, which leads to cognitive defects (103,210). The DNA of noradrenaline neurons is more liable to oxidative damage than others, which is a main concern in patients with OH (211).

Noradrenaline markers are reduced to a high degree in patients with both PD and dementia (212). Depending on the available data, noradrenaline can be used as a parameter for cognitive affection in various neuronal diseases, such as Parkinsonism (213). The density and volume of the basal forebrain cholinergic area (the main source of acetylcholine innervation to the amygdala, neocortex and hippocampus) are reduced in PD in both newly diagnosed patients and during the course of the disease (214-216). This area is crucial for cognition, particularly memory, attention and execution (217,218). In patients with dementia and PD, the loss is mainly in cholinergic fibers, not neurons (219). The loss of acetylcholine innervation to the cortex independently leads to a decreased cognitive level. This is associated with dopamine loss and further cognitive defects (215,220). Dopamine terminals heavily innervate the cholinergic area of the forebrain, which may cause more cognitive defects in these cases (221). In addition, the loss of cholinergic innervation from the forebrain to the hippocampus leads to memory troubles and deterioration to dementia (216,222). In cases of PD with MCI, there is a decrease in acetylcholine fibers in the hippocampus and their activity. Still, in cases having both PD and dementia, there is also the deposition of α-synuclein in the hippocampus and forebrain basally (223,224). The mechanisms through which the cholinergic system in the basal forebrain degenerates have not yet been fully elucidated. The noradrenergic system in locus coeruleus is more exposed to oxidative damage than cholinergic neurons (211). Following the decrease in acetylcholine fibers in the cortex, α-synuclein is widely and non-non-specifically aggregated in multiple neurons and neurotransmitters of variable types (224). Fibers that contain galanin increasingly innervate the cholinergic neurons in the forebrain, basically in PD cases at the time of MCI development and progression to dementia, which may be a cell response to injury that occurs following α-synuclein aggregation (219).

Although serotonergic neurons are lost early from the brainstem and even before the loss of dopaminergic neurons, there is no definite association between the loss of serotonin neurons and cognitive defects in PD (225). Serotonin loss is associated with defects in motor and some non-motor functions, such as anxiety, depression and sleep disorders (226,227). The loss of serotonin from the brain in PD is likely due to β-amyloid deposition, and drugs that increase serotonin transmission decrease β-amyloid and cognitive defect (202). In PDD, α-synuclein deposition is the cause of cognitive affection and other age-related pathologies (228). Inflammation of the neurons occurs only when Lewy pathology is associated with AD (229). In the majority of cases of PDD, the limbic system, with or without the neocortex is affected by Lewy pathology, but some cases do not have the same pathology (149). In mild cognitive impairment in Parkinson's disease (PD-MCI), the entorhinal cortex is atrophied, which affects memory (230). α-synuclein deposition in this region leads to disease progression to dementia (231). It has been shown that α-synuclein deposition in the neocortex is the main cause of PDD (149). Furthermore, α-synuclein affects the DNA of the neuron and its repair (232). Of note, SNCA (the gene coding for α-synuclein) is genetically variable and differs between PD and DLB. Whether or not α-synuclein levels can predict cognitive defect levels remains unclear (202).

Patients with PD and cognitive affection are likely to have depositions of β-amyloid extracellular and tau intracellular (aging pathology that occurs in AD) (147,150,233). The deposition of β-amyloid precedes tau deposition and together leads to AD (234).

A number of cases of PDD have positive PET results (β-amyloid PET) (233). When AD is associated with PD, this increases the amount of α-synuclein deposition and suggests progression to PDD (149,235). In addition, these two diseases together exhibit more inflammation of neurons and amyloid deposition with a significant defect in cognition (149). Furthermore, these patients usually suffer from language impairment more than those having PD only without AD, with more tau deposition (236). It has been noted that TDP43 and cerebrovascular pathology do not play any role in PDD (149).

Genetics plays a crucial role in general and PD cognition (237). Genetics can affect α-synuclein metabolism (in lysosome), potassium channels, such as Transmembrane Protein 175 (TMEM175), and α-synuclein levels and may increase α-synuclein in PDD (238,239). Mutations in glucocerebrosidase beta acid (GBA), SNCA and TMEM175 (that encode β-glucosylceramidase) reduce potassium current and elevate α-synuclein levels, thus impairing mitochondria and lysosomal function along with a decrease in lysosome activity and glucocerebrosidase with an increase in the risk for PD and PDD (240).

The decreased activated glucocerebrosidase and low potassium level increase α-synuclein phosphorylation and subsequent cell pathology (240). A certain polymorphism in the nucleotide of GBA decreases the expression of glucocerebrosidase and increases α-synuclein deposition, leading to PDD (241). APOE ε4 allele that encodes APOE increases the cognitive defect (237). This allele may also increase the deposition of β-amyloid in normal and diseased populations. Genetic changes in SLC6A3 (or DAT that encodes for the transporter of dopamine) are associated with poor cognition and reduced dopamine transmission (242). There are numerous genes that can affect cognition, such as those concerning dopamine synthesis (DDC), degradation (COMT encodes for catecholamine-*O*-methyltransferase) and receptors of dopamine (DRD2, encodes for receptor 2 of dopamine) (243). Variations in the levels of these genes may lead to cognitive disorders in PD (202).

## 4. Dysautonomia as a risk factor for cognitive impairment

The pathology of PD has a wide range of neuronal affection that extends outside the dopaminergic nigrostriatal system, leading to a number of non-motor issues. The accumulation of Lewy particles in the neocortex and limbic system is considered to be the cause of dementia and cognitive impairment in PD (244). However, to date, studies have not found a clear connection between the amount of Lewy particles in the cortex and dementia in Parkinsonism (245). Similarly, Lewy particle accumulation inside autonomic neurons peripherally and in sympathetic ganglions may be associated with OH (autonomic dysfunction) (246). Central components of the autonomic system may also be involved, particularly the hypothalamus, intermediolateral nucleus (in the spinal cord), and vagus nucleus (dorsal part) (247). According to the study by Kosaka (248), different disorders occur due to Lewy molecules. The existence of these molecules and their distribution in the brains of deceased individuals has helped scientists to find certain criteria for each syndrome. Researchers have found a variable connection between autonomic defects, parkinsonism and dementia (249). When Lewy bodies are present only in the brainstem, this leads to idiopathic parkinsonism; however, when these bodies are present widely in the cortex beside the brainstem, this leads to PDD. The accumulation of these bodies in the sympathetic ganglion, spinal cord and brain stem may lead to OH and parkinsonism (249) Previously, Braak *et al* (250) extended this concept using the stagewise hypothesis for synuclein progression in idiopathic parkinsonism.

According to the available information, synuclein is deposited extra-nigrally, firstly in the vagus nucleus (dorsally) and the olfactory system, then nigral disorders in the third stage, and finally in the cortex. This explanation does not include the peripheral neurons (of the autonomic system) or the spinal cord. However, it has been proposed that the efferent sympathetic supply to the heart and intermediolateral nucleus are the first sites to deposit Lewy particles in non-symptomatic patients with PD (246). Thus, perhaps the autonomic system plays a role in those having Lewy particles in their cortex and complaining of dementia. Horimoto *et al* (251) reported autonomic issues in all deceased individuals who had Lewy particles along with dementia in their study. These issues were variable from OH, constipation and urinary incontinence. Orimo *et al* (252) also reported the loss of sympathetic innervation to the heart in all DLB cases in their study In addition, OH has been noted to occur more among PDD compared to those without dementia. Larner *et al* (253) also suggested that synuclein is deposited firstly in the autonomic system and then in the cortex after years.

Due to aging of the vascular and autonomic nerve systems, as well as a decrease in baroreceptor sensitivity, OH is common in the elderly (254). Furthermore, any abrupt change in BP that causes a rapid and considerable shift in cerebral blood flow may be considered to produce or exacerbate cognitive impairment as aging is linked to impaired cerebral autoregulation (255). The association between cognitive impairment and OH in the elderly is presented in Table III. No marked differences were reported in cognitive function and MMSE between individuals with OH, and individuals without OH.

## 5. Pathophysiology of dysautonomia and cognitive impairment

Autonomic dysregulation occurring in PD occurs by both central and peripheral pathways (256). Aggregates of α-synuclein protein forming Lewy bodies along with neuron loss are observed in the zona compacta of the substantia nigra and the regions controlling the autonomic functions (257).

Normally, baroreceptors maintain BP during standing by releasing noradrenaline from the postganglionic neurons of the sympathetic nervous system (104). However, this mechanism is impaired in PD due to degeneration of the postganglionic component of the sympathetic nervous system (the main cause of dysautonomia affecting the heart in PD) (104,258). In PD, the cutting of sympathetic innervation to the heart can be shown using cardiac MIBG scintigraphy, which appears to have a low myocardial uptake, and neuropathological imaging shows fiber loss (259). Lewy bodies are characteristic of neuropathology in PD and present in the cardiac plexus of the affected individuals (260). In addition, sympathetic denervation to the extracardiac blood vessels occurs, affecting both noradrenaline release and vasoconstriction during standing (258). Normally, the noradrenaline level is doubled during standing after almost 5 min; however, this level appears to be decreased in patients with PD-OH than in those without OH (166,261). A progressive decrease in BP in phase two of the VM in nOH, along with the loss of overshooting in BP during phase four, suggests the loss of sympathetic baroreflex, which may lead to systolic hypertension (258). nOH can result in a weak change in the heart rate when changing positions from supine or standing. The differentiation between nOH and non-nOH can be achieved by monitoring the increase in heart rate and the fall in systolic BP. nOH is associated with a mild increase in the heart rate and a gain in baroreceptors by less than five beats per minute/mmHg (116,262). In addition, Tipre and Goldstein (263) noted a decrease in the sympathetic innervation to the kidney with blood volume depletion due to diuresis and natriuresis. The management of PD mainly involves the use of levodopa alone or along with benserazide or carbidopa, leading to an increase in dopamine levels and its metabolites in the plasma. This leads to vasodilation along with an increase in natriuresis and diuresis, leading to a reduction of both extracellular fluid and blood volume (258). The impairment of the sympathetic innervation to the heart and baroreflex in PD leads to hypotension (104). Furthermore, Noack *et al* (264) presumed that the negative inotropic effect that occurred with levodopa was the cause of hypotension, not vasodilatation. In PD, the relation between manifestations of dysautonomia and involved structures of the autonomic nervous system remains unclear (256). The central biological clock and the hypothalamus regulate the circadian rhythm of heart rate and BP through the suprachiasmatic nucleus (SCN) (265). This nucleus activates the autonomic innervation through both GABA neurons and the regulation of the release of melatonin (265). A previous study found that α-synuclein is deposited in the SCN of affected individuals (257). However, another study found a low serum melatonin level in the affected people (266). In the brain, noradrenaline is mainly produced by the nucleus coeruleus, which is early affected in PD (267) along with a decrease in the transporter of noradrenaline (209).

A reduced number of catecholamine neurons in the solitary tract may cause impaired baroreflex (268). In patients with combined PD and OH, an increased level of Lewy bodies in the cerebral cortex (insular part) is noted with a defect in the functional connection between the striatum, thalamus and hypothalamus (269,270).

WMHs are regions of the heightened signal detected on T2-weighted or fluid-attenuated inversion recovery (FLAIR) magnetic resonance images. These hyperintensities primarily arise from long-term, widespread and asymptomatic ischemia (i.e., reduced blood flow to brain tissue, leading to insufficient oxygen and glucose supply and disrupting cellular metabolism). While periventricular regions are primarily affected, WMHs can occur throughout the entire brain (271,272). WMHs are commonly observed in elderly individuals, as well as in individuals diagnosed with AD. These WMHs are indicative of the existence of demyelination and axonal degradation (272). WMHs can have a significant effect on the cognitive function of both healthy older patients, and patients with MCI and dementia (273,274). In PD, WMHs have been linked to OH (35,120). The association between the WMHs and OH supports the theory that repeated episodes of hypotension may result in reduced blood flow to the brain, potentially causing damage to susceptible brain regions and cognitive impairments (103). Moreover, the degenerative mechanisms associated with age and PD may contribute to the changes in white matter, possibly due to vascular insufficiency (120).

Early WMHs have also been associated with cognitive impairments and a subsequent higher likelihood of developing dementia in individuals with PD (275,276). Examining the temporal linkages between dysautonomia, WMHs and cognitive decline could provide insight into the fundamental mechanisms of these significant non-motor symptoms in PD.

**Figure 2:**
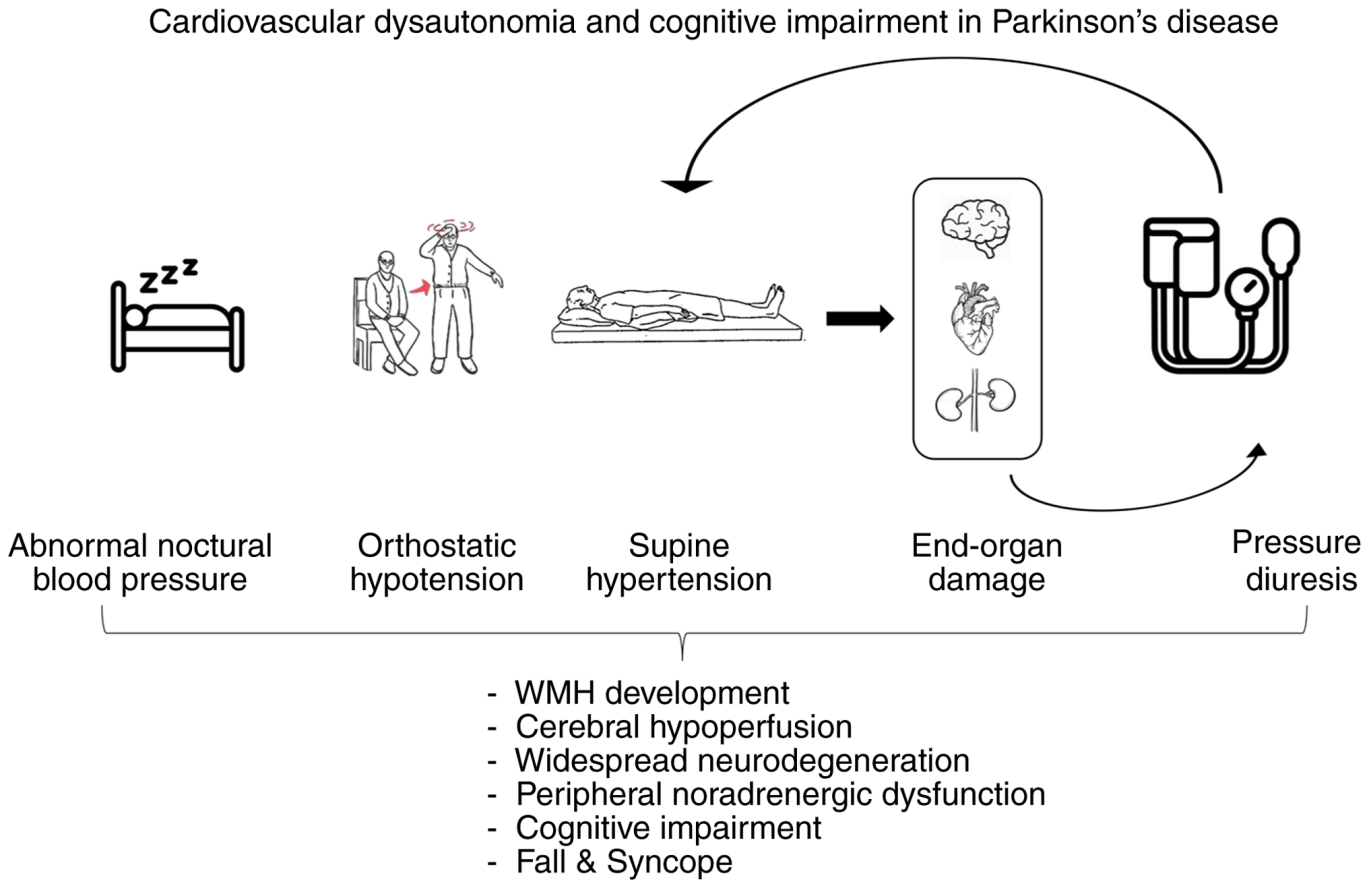
Pathophysiology of autonomic dysfunction and cognitive impairment in PD. WMHs are associated with diastolic OH and partially explain the impact of autonomic dysregulation on cognitive loss in patients with PD. Autonomic dysfunction in the initial stages of clinical development makes the brain more susceptible to WMHs by disrupting blood flow in the small blood capillaries. SH can result in kidney damage, which in turn causes pressure diuresis and aggravates OH. Therefore, a cycle leading to the development of cognitive abnormalities can be noticed. PD, Parkinson's disease; WMHs, white matter hyperintensities; OH, orthostatic hypotension; SH, supine hypertension.

Furthermore, studies have revealed that in patients with PD and other synucleinopathies, such as multiple system atrophy, dementia with Lewy bodies and pure autonomic failure, SH plays a crucial role. Specifically, the average BP measurement in a supine position was the most reliable indicator of target organ damage. Additionally, SH was independently associated with a greater burden of WMHs, along with more severe renal failure and a higher prevalence of left ventricular hypertrophy (118). Similarly, within the overall population, experiencing reverse dipping (nocturnal hypertension) is significantly linked to an increased risk of developing cerebral small vessel disease and cognitive impairment (277). Cerebral small vessel disease is the primary cause of vascular dementia, encompassing lacunar infarcts (LCI) and WMHs (278). Hypertension significantly increases the likelihood of experiencing cognitive impairment, and there is a clear association between reduced brain blood volume, neuritic plaques and hypertension (279). Earlier onset and more severe manifestations of OH are associated with an increased risk of developing dementia in patients diagnosed with PD. Some authors have hypothesized that individuals with earlier and more severe OH will exhibit more severe brain disorders characterized by the accumulation of abnormal proteins and/or abnormalities in the blood vessels, particularly in the presence of concomitant SH (280). Due to the simultaneous occurrence of OH and SH in the same patient, separating and understanding their individual implications is challenging. OH has both immediate, brief effects, such as fainting and accidents, as well as long-term issues such as kidney failure (41). The difficulty in distinguishing the effects of OH from those of SH leads to conflicting opinions among clinicians regarding the optimal approach to managing both conditions, as addressing one condition typically worsens the other (31,104). A schematic summary of the pathophysiology of autonomic dysfunction and cognitive impairment in PD is presented in Fig. 2.

## 6. Treatment of cardiovascular dysautonomia

### nOH

**Figure 3:**
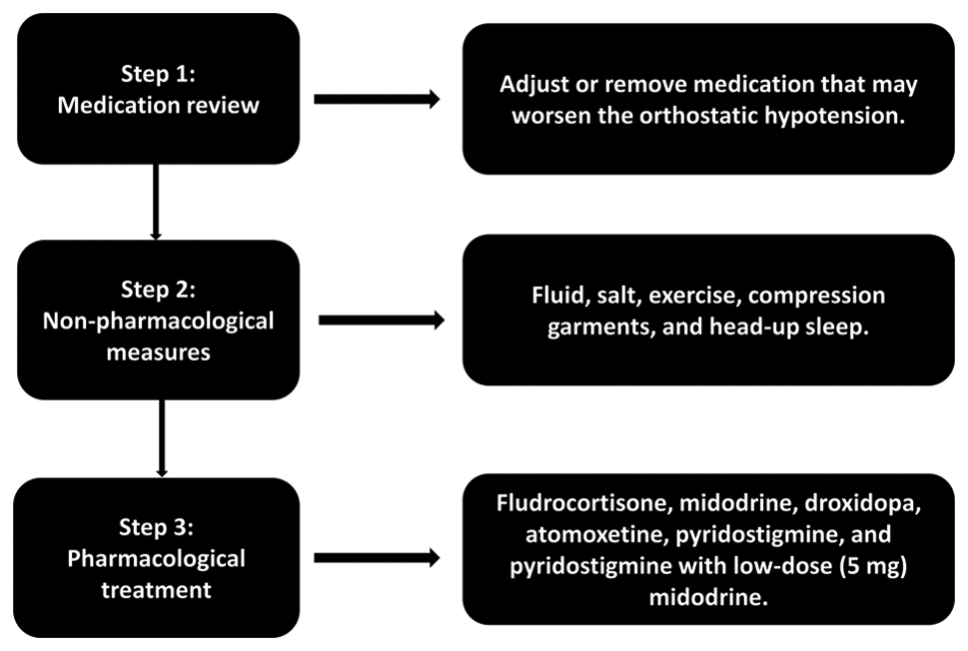
Management of OH. After diagnosing the case as an OH, there are three steps that should be followed to manage the case. Pharmacological simplification should be considered by reducing or terminating medications that exacerbate nOH. Subsequently, the non-pharmacological measures should be started before using medications to treat nOH. Finally, pharmacological treatment is still needed in many cases to alleviate symptomatic nOH. OH, orthostatic hypotension; nOH, neuxrogenic orthostatic hypotension.

The primary goal of treating patients with nOH with synucleinopathies is not to achieve a normal standing BP, but rather to decrease the severity of symptoms, enhance the quality of life, and diminish the risk of complications and mortality (281). There are established criteria for the management of nOH. If nOH does not exhibit any symptoms, treatment may not be necessary or may be confined to non-pharmacological approaches. Pharmacological treatment is typically necessary when nOH is symptomatic, causing symptoms of organ hypoperfusion, such as dizziness, lightheadedness and blurred vision (32,262). The management flowchart of OH is illustrated in Fig. 3.

*Reviewing and adjusting current medications*. After receiving a clinical diagnosis of nOH, a patient should immediately consider pharmacological simplification by reducing or stopping medications that exacerbate nOH. It is essential to review all medications carefully to make necessary schedule adjustments during the first few weeks of treatment. A number of medications, including those broadly used to treat PD, hypertension or bladder issues, can bring down the pulse and exacerbate nOH. Some patients may be able to combat their nOH symptoms by stopping or decreasing the dose of medications, such as diuretics and vasodilators, as well as those with negative chronotropic effects, such as β-blockers (262). Anemia requires investigation and necessitates treatment (282). The administration of erythropoietin, along with iron supplements, may provide improvements for those with nOH and anemia (283).

*Non-pharmacological measures*. The recommended daily fluid consumption is between 2 and 2.5 liters. Patients should be advised to increase their salt consumption by incorporating 1-2 teaspoons of salt into a nutritious meal. Some patients prefer using 0.5-1.0 g salt tablets despite their potential to cause gastrointestinal discomfort. Patients with nOH exhibit a significantly increase in BP after consuming 0.5 liters of water (284). Patients should pay attention to the diuretic properties of coffee and alcohol and avoid consuming sugary drinks, such as bottled juices and sodas, as these may cause a drop in BP (285). Symptomatic nOH can rapidly result in a disinclination to rise and a tendency to avoid engaging in physical exertion.

Physical immobility exacerbates OH (286). Physical exercise is an essential part of the treatment plan. However, it should be noted that performing physical activity while standing may exacerbate hypotension in patients with autonomic failure. The recommended position for exercise is either recumbent or sitting, using a recumbent stationary bicycle or rowing machine (287). Food intake leads to the accumulation of blood in the splanchnic circulation, causing individuals to have significantly low BP within 2 h of eating. This condition is known as PPH and is most common after meals high in carbohydrates. Consuming smaller and more frequent meals, as well as decreasing the intake of carbohydrates, can enhance the condition of PPH (33,58,288).

Syncope is often caused by straining and Valsalva-like maneuvers during bowel movements (289). In this situation, the treatment of constipation is necessary (290). Compression garments can prevent syncope and should be considered (291,292). Sleeping with the head up also has a potential benefit in the management of nOH (291).

*Pharmacological treatment*. Despite the effective implementation of non-pharmacological approaches, a significant number of patients still necessitate pharmacological treatment to alleviate symptomatic nOH (104). One approach is to increase the vascular tone using sympathomimetic drugs, while another is to augment the circulating blood volume by raising the plasma or red cell mass (293). Many physicians consider that it is reasonable to start medication at the beginning of management for patients who are having syncope, near-syncope, or falls due to the serious possible implications. Clinicians must customize the treatment plan according to the severity and urgency of the symptoms (262). The choice between one or both options is determined by the individual characteristics and requirements of each patient, as well as the extent of peripheral sympathetic denervation (104).

Fludrocortisone, also known as 9α-fludrocortisone, is an artificial mineralocorticoid that raises BP through at least one of the following pathways: It promotes sodium and water reabsorption in the kidneys, increasing fluid volume within blood vessels. Additionally, it improves the ability of the body to respond to natural stress hormones and medicines that increase BP (294). However, fludrocortisone worsens sodium and water retention and contributes to the progression of left ventricular hypertrophy and renal failure, potentially increasing the likelihood of hospitalization for any reason (295). Other common side-effects include hypokalemia and ankle edema (294,296). Fludrocortisone is usually prescribed at a daily dosage of 0.1-0.2 mg, and no significant improvement has been reported by increasing the dose to >0.3 mg per day. In fact, increasing the dose beyond 0.3 mg per day is associated with an elevated risk of side-effects (297-300).

Another key pharmacological class for the management of nOH is the pressor agents. Midodrine is a type of medication that is converted into a substance known as desglymidodrine in the body. Desglymidodrine acts as a stimulant for a specific type of receptor termed α1-adrenoreceptor, which helps increase blood vessel resistance and BP. The recommended dose ranges from 2.5 to 15 mg, taken once to thrice daily during waking hours. For instance, a three-times daily plan could involve taking the medication before bed, lunch and mid-afternoon (301). The administration of midodrine leads to a notable elevation in both systolic and diastolic BP, accompanied by slight enhancements in orthostatic symptoms. Midodrine poses a notable risk of causing SH; thus, it is advised that patients refrain from taking midodrine within 5 h of going to bed (28,302,303). Stimulating α1-adrenergic receptors can lead to undesirable effects, including piloerection (often known as ‘goosebumps’), itching of the scalp and urine retention. Midodrine does not affect the heart rate, since it does not stimulate β-adrenoreceptors. Additionally, it has no deleterious effects on the central nervous system due to its limited ability to penetrate the blood-brain barrier (304).

Droxidopa, also known as L-threo-3,4-dihydroxyphenyl-serine or L-DOPS, is an orally administered synthetic amino acid that undergoes conversion to norepinephrine within the body (305). The maximum levels of droxidopa in the bloodstream are achieved within ~3 h following its oral administration. The amount administered in clinical studies ranged from 100 to 600 mg, taken three times per day. However, clinical observations suggest that the dosage should be individualized based on the specific needs of each patient, taking into account their periods of activity and inactivity (305-307). Due to the variability in the pressure impact of droxidopa across patients, it is strongly advised to undergo a titration procedure under the supervision of a clinician (281). Additional research is required to determine whether droxidopa has positive effects on both motor and non-motor symptoms caused by a lack of norepinephrine in patients with PD (267). The FDA has approved atomoxetine, a medication that selectively blocks the norepinephrine transporter, for the treatment of attention deficit hyperactivity disorder (308). Prior research has demonstrated that administering a daily dosage of 18 mg of atomoxetine effectively alleviates OH and symptoms associated with nOH in patients (309,310). Following atomoxetine delivery, elevated norepinephrine levels have been linked to an elevated standing BP (311). Autonomic dysfunction is also observed in patients with drug-induced parkinsonism, and atomoxetine has shown promise in treating drug-induced parkinsonism, particularly in patients whose elevated sympathetic tone is a symptom of a psychiatric disorder (30,312)

In patients with nOH, either pyridostigmine alone or combined with low-dose (5 mg) midodrine hydrochloride will improve orthostatic BP without aggravating SH (313). However, the combination of 5 mg midodrine and 60 mg pyridostigmine has exhibited better results than either agent used alone. Pyridostigmine is less effective than midodrine in alleviating symptoms linked to nOH (314). Case reports and proof of concept studies have demonstrated the effectiveness of yohimbine, ergotamine, dihydroergotamine, ephedrine, desmopressin, indomethacin and fluoxetine in the treatment of nOH (315).

### Neurogenic SH (nSH)

Various non-pharmacological approaches can be employed to decrease nOH. Information about these methods should be included in the education of patient on nOH (262,316). Supine BP can be reduced by raising the head of the bed (111,317). Patients should raise their heads off the beds 6 to 9 inches (~30˚) when sleeping at night. Sleeping with the head up lowers BP and natriuresis, while maintaining an activated renin-angiotensin system, resulting in a less abrupt drop in BP upon waking (299). Another strategy for lowering nocturnal hypertension is to consume a snack high in carbohydrates before going to bed. This has two effects: First, it reduces BP by transferring blood to the splanchnic circulation; second, it triggers insulin release, which has a direct vasodilator effect (305,318). Reducing BP can also be achieved with the aid of local passive heat. Patients suffering from SH have had their BP reduced overnight by using a heating pad set at 40-42˚C for 2 h (319). Using continuous positive airway pressure (8-12 cm H_2_O) overnight is a novel non-pharmacological method for treating SH. This method reduced BP throughout the night, reduced diuresis during the night, and improved morning symptoms of nOH (320).

A short-acting antihypertensive medication taken before bed may be required if nSH continues to be present after nonpharmacologic methods have been exhausted (321,322). In general, patients with severe nSH (systolic/diastolic BP ≥180/≥110 mmHg) may require the pharmacological management of nSH. By contrast, those with moderate nSH (BP 160-179/100-109 mmHg) may be considered for pharmacological care, depending on their specific case and risk profile (116,262). When possible, it is best to use short-acting medications to treat hypertension so that the symptoms of patients with nOH do not worsen upon waking in the morning (322). Medications that can be used to treat SH include captopril, eplerenone, hydralazine, losartan, nifedipine and nitroglycerin patches, as summarized in Table IV.

## 7. Treatment of cognitive impairment

### Treatment of risk factors and cognitive training intervention

The factors that can worsen cognitive impairment need to be assessed and resolved. Polypharmacy, mental issues and sleep disturbances are all examples of such conditions (323,324). When feasible, it is best to avoid psychoactive substances. Medications with minimal negative effects on cognition should be evaluated when treatment is required.

As regards treating depression in PD, for instance, paroxetine is effective. However, patients with PD who also have cognitive impairment should not take paroxetine, since it has the worst anticholinergic profile of all the depression drugs (325,326). At each visit, it is critical to check for medications with direct and indirect anticholinergic effects. Patients with PD should not be initially prescribed the anticholinergic medicine oxybutynin for the treatment of overactive bladder as it has been demonstrated to reach the central nervous system and induce cognitive impairment in certain individuals (327). It is also critical to check for non-prescription drugs that can cause side-effects, such as diphenhydramine-containing over-the-counter sleep aids (328). Patients should be advised to stop using these products and instead try sleep hygiene techniques that do not involve medication or sleep aids that target both sleep and any other symptoms that may be present, such as depression, psychosis or hallucinations (329). Cognitive-based therapies are distinct from non-pharmacological interventions, since they aim to improve cognition rather than other behavioral or functional objectives, including cognitive stimulation, training, and rehabilitation (330). Cognitive stimulation provides broader stimulation to enhance cognitive and social performance. Cognitive training employs standardized cognitive tasks administered through computer-based or paper-based methods. Cognitive rehabilitation focuses on addressing specific challenges in everyday tasks to enhance overall functioning (330). Various physical workouts, such as treadmill training, dance, stationary cycling, Wii Fit and Tai Chi, have been assessed for their impact on cognition. An extensive analysis of randomized controlled trials (RCTs) examining the impact of physical exercise on cognition in individuals with PD, including those with normal cognition and PD-MCI, revealed that physical exercise resulted in enhancements in overall cognitive function, processing speed, attention and mental flexibility. Utilizing the treadmill three times per week for 60 min was shown to result in the most significant improvement in cognition (331). For those with mild to severe PD with a MMSE score >24, a regimen of treadmill walking consisting of 45 min per session, 3 days per week, for a duration of 3 weeks was shown to lead to a substantial enhancement in executive function as assessed by the FAB, trail-making and memory interference tests (332).

Transcranial magnetic stimulation has been studied in the context of PD for the treatment of motor, emotional and cognitive symptoms. There are insufficient conclusive data to support the effectiveness of repetitive transcranial magnetic stimulation in enhancing cognitive function in individuals with cognitive impairment associated with PD. A previous study demonstrated that repetitive intermittent stimulation, known as ‘theta burst’ of the left dorsolateral prefrontal cortex (DLPFC) resulted in enhanced cognitive abilities and visuospatial ability that persisted for up to 1 month in individuals with PD-MCI (333). Transcranial direct current stimulation (tDCS) applied to the prefrontal cortex has been shown to result in enhanced executive function, namely in trail-making activities. Still, it has not led to similar improvements in other tests, such as the Stroop test or the Wisconsin Card Sorting Test, in patients with PD who did not have dementia (334). An RCT with a sample size of 22 participants demonstrated that the combination of cognitive training and tDCS applied to the left DLPFC in adults with PD-MCI resulted in improved phonemic verbal fluency compared to cognitive training alone. This improvement was shown following a treatment period of 5 days per week for 2 weeks and was sustained at the 3-month follow-up assessment. Nevertheless, there were no discernible variations between the groups regarding other key variables related to language, attention, and executive processes (335).

### Pharmaceutical management of PDD

The precise mechanisms responsible for the cognitive decline in PD remain incompletely known. It is widely recognized that patients with PD experience the early degeneration of cholinergic neurons in the basal forebrain. These neurons supply cholinergic connections to the neocortex (247,336). As a result, the use of acetylcholinesterase inhibitors (AChEI) has been thoroughly examined for the treatment of PDD. Rivastigmine is an AChEI, one of the three AChEIs approved by the FDA for the treatment of cognitive symptoms in AD. It possesses a distinct characteristic of inhibiting acetylcholinesterase and butyrylcholinesterase, which has been proposed as a possible explanation for its effectiveness (337). The efficacy of rivastigmine was established in a 24-week clinical trial with 541 individuals with mild to moderate PDD. The trial was conducted double-blind with a placebo control group (3). The doses of the study medicine were gradually increased over 16 weeks, with each patient being maintained on the greatest dose they could tolerate for the remainder of the study. The average dosage of rivastigmine reached 8.6 mg per day by the conclusion of the dose-escalation phase. The main measures of effectiveness were the scores for the Alzheimer Disease Assessment Scale-Cognitive Subscale (ADAS-cog) and the Alzheimer's Disease Cooperative Study-Clinician's Global Impression of Change (ADCS-CGIC). The secondary outcomes included the Alzheimer's Disease Cooperative Study-Activities of Daily Living (ADCS-ADL), the 10-item neuropsychiatric inventory, the MMSE, the cognitive drug research computerized assessment system, the Delis-Kaplan Executive Function System (D-KEFS) Verbal Fluency Test, and the 10-point Clock Drawing Test. The rivastigmine group demonstrated a mean improvement of 2.1 points on the ADAS-cog after 24 weeks of treatment, while the placebo group experienced a decrease of 0.7 points. There was a 19.8% clinical improvement on the ADCS-CGIC in the group treated with rivastigmine, compared to a 14.5% improvement in the group treated with placebo (P=0.007). In addition, a significant increase in clinically significant deterioration on the ADCS-CGIC scale was detected in 13% of the participants in the rivastigmine group and 23.1% in the placebo group (P=0.007). Furthermore, rivastigmine demonstrated a substantial advantage over placebo in all additional measures of effectiveness. It is worth mentioning that a somewhat higher number of patients in the rivastigmine group reported experiencing symptoms such as nausea, vomiting, tremors, and dizziness (3). Rivastigmine has the potential to enhance apathy in PD, in addition to its effects on cognitive impairment. A 6-month clinical experiment was conducted to evaluate the effects of a rivastigmine transdermal patch on apathy in 30 patients with PD who did not have dementia or depression. The trial was double-blind, placebo-controlled, and randomized. The results showed that the rivastigmine transdermal patch substantially impacted the Lille Apathy Rating Scale (LARS) in patients with moderate to severe apathy (338). Other treatment options, including donepezil, galantamine, and memantine are summarized in Table V.

The current guidelines for the management of arterial hypertension provide a comprehensive approach to treating patients with comorbid conditions such as PD (44). Studies on cholinesterase inhibitors and memantine have shown varied efficacy in improving cognitive impairment in patients with PDD and dementia with Lewy bodies (339-341). Furthermore, treatment with cholinesterase inhibitors like donepezil has demonstrated efficacy in clinical trials, specifically in patients with PD dementia (342). Research also indicates that certain interventions targeting orthostatic hypotension and syncope in Parkinson's disease can have positive effects on overall cognitive and functional outcomes (343-347).

In addition, the treatment of autonomic dysfunctions with pharmacological agents, such as midodrine and droxidopa has been evaluated, with findings supporting their use in managing orthostatic hypotension and its associated cognitive decline (348-353).

## 8. Future studies

In recent years, notable achievements have been made, such as the commencement and successful conclusion of extensive clinical trials and the authorization of novel treatments for autonomic dysfunction and cognitive impairment, each one separately. However, investigating the association between cardiovascular dysautonomia and cognition in PD can provide a clearer understanding of the underlying mechanisms that connect these significant nonmotor symptoms. This includes examining the pathological and neurochemical connections.

Cognitive impairment may be directly and indirectly related to autonomic dysfunctions. Further studies about the pathophysiology of cognitive function in individuals with PD are warranted, particularly as it affects 4 out of every 5 individuals with PD. The current literature shows an indirect association in which patients with autonomic dysfunctions have been observed to have white matter changes (280). It is worth investigating the cardiac function with cardiac MIBG before developing peripheral symptoms and then evaluating neuroimaging with different sequences and volumetric studies. It is possible that the occurrence of autonomic symptoms is directly associated with the progression of the disease, and at the same stage, the patient develops independent cognitive dysfunction. Notably, some studies have revealed improved cognitive function with the optimized management of cardiovascular dysfunction (89-91).

Conducting larger prospective studies that analyze clinical, genetic, and pathological data will provide a better understanding of the variability in autonomic and cognitive impairment in PD. Moreover, it is necessary to conduct clinical trials to examine whether dealing with dysautonomia during the prodromal or early stages will decelerate or delay cognitive deterioration. Additional information about the link between dysautonomia and cognitive impairment may be revealed by functional imaging, which might be combined with new magnetic resonance imaging methods to assess connectivity. Understanding the rate and pattern of cognitive decline becomes essential for future research, as it varies significantly. Investigating the underlying mechanisms behind various clinical patterns is an important priority.

## 9. Conclusions and future perspectives

In summary, the present review described a significant association between cardiovascular dysautonomia, in particular OH, and cognitive impairment. Dysautonomia, WMHs and cognitive decline are major non-motor symptoms in PD, and understanding their temporal and causal linkages may shed light onto some of the underlying pathophysiological mechanisms. It is a multifactorial process, and the most commonly suggested mechanisms are hypoperfusion in the white matter of the brain or the spread of α-synuclein protein from the peripheral autonomic nervous system to the central nervous system, recurrent episodic brain hypoxia/ischemia, which is linked to widespread neuronal apoptosis and significant infarction. These findings imply that repeated episodes of reduced blood flow and oxygen supply to the brain caused by dysautonomia may lead to a higher likelihood of significant accumulation of α-synuclein protein and, therefore, be linked to cognitive loss. Thus, it is a vicious cycle and linked mechanisms that finally lead to the development of this association. If dysautonomia is identified as a potential modifiable risk factor for cognitive impairment, then early diagnosis and treatment would be an essential approach for minimizing the risk of future cognitive decline. Hence, treating BP dysregulation may impede the advancement of cognitive deterioration in PD. Further clinical trials with a larger population are required to confirm these findings on the pathogenesis of cognitive impairment in the very early stages of PD, which may be prevented.

## Figures and Tables

**Table I:** Summary of different patterns of BP dysregulation associated with PD.

Disorder	Definition	Authors/(Refs.)
OH	Decrease in systolic BP ≥20 mmHg or decrease in diastolic BP ≥10 mmHg when standing or tilting the head up to at least 60˚ within 3 min.	Lahrmann *et al* (32)
SH	Systolic BP ≥150 mmHg or a diastolic BP ≥90 mmHg.	Goldstein *et al* (39)
Nocturnal BP abnormalities	Values >125/75 mmHg are considered abnormal.	Pickering *et al* (43), Mancia *et al* (44), Ogihara *et al* (45)
Postprandial hypotension	Decrease in systolic BP ≥20 mmHg within a time frame of 2 h after consuming a meal.	Umehara *et al* (57)
Post-exercise hypotension	Temporary BP drop after an acute exercise bout. The amplitude and duration of this post-exercise BP decline vary among different studies.	Low *et al* (61)

PD, Parkinson's disease; BP, blood pressure; OH, orthostatic hypotension; SH, supine hypertension.

**Table II:** Comparison between PD and dementia with Lewy bodies.

Aspect	PD	Dementia with Lewy bodies
Age	Young age	Elderly
Sex distribution	More frequently female	More commonly male
Unified Parkinson's disease rating scale motor scores	No significant difference	No significant difference
Olfaction impairment	Less severe	More severe
Mini-mental state examination scores	Lower	Lower
Frontal assessment battery scores	Lower	Lower
Cardiac MIBG uptake (H/M ratio)	Reduced	Reduced
OH	Less prevalent	More prevalent
Nocturnal BP fall	Less pronounced	Reduced
Non-dipper/riser types	Lower percentage	Higher percentage
Postprandial hypotension	Less pronounced	Larger fall and prevalence
SH	No significant difference	Higher prevalence (neurogenic)
Constipation	Less prevalent	More prevalent

PD, Parkinson's disease; BP, blood pressure; MIBG, 123I-metaiodobenzylguanidine; OH, orthostatic hypotension; SH, supine hypertension.

**Table III:** Cognitive impairment and OH in the elderly.

	N^a^		Sex^b^		Age^c^		Education^d^		MMSE		Cognitive impairment		
Authors	OH (+)	OH (-)	OH (+)	OH (-)	OH (+)	OH (-)	OH (+)	OH (-)	OH (+)	OH (-)	OH (+)	OH (-)	(Refs.)
Viramo *et al*	931	228	592	NA	76.0 (±4.9)	76.0 (±5.1)	NA	NA	21.6 (±3.9)	21.1 (±4.0)	NA	NA	(339)
Kuo *et al*	70		NA	NA	72.0 (± 4.0)		NA	NA	NA	NA	NA	NA	(340)
Allcock *et al*	42	45	NA	NA	72.6 (±8.1)	68.2 (±9.6)	NA	NA	NSS		NSS		(341)
Yap *et al*	381	1,913	259	249	66.6 (±8.5)	65.3 (±7.1)	59.1	49.9	26.7 (±3.5)	27.3 (±3.2)	16.6	9.9	(342)
Bendini *et al*	9	27	10		80.5 (±6.2)		NA	NA	21.3 (±4.8)		NA	NA	(343)
Rose *et al*	652	12,050	364	10,801	57.3	53.9	32.4	22.4	NA	NA	NA	NA	(344)

^a^Total number of individuals enrolled in the study;

^b^number of individuals of the female sex;

^c^mean age of the individuals and the standard deviation;

^d^percentage of individuals with less than six years of formal education. NA, not available/not reported; NSS, no statistical significance; OH, orthostatic hypotension; OH (+) individuals with OH; OH (-) individuals without OH.

**Table IV:** Summary of pharmacological treatment of nOH and SH.

A, nOH				
Drug	Mechanism	Recommendations	Adverse effects	Authors/(Refs.)
Atomoxetine	Norepinephrine transporter blocker.	Initially, 10 mg twice daily; this may increase to 18 mg twice daily.	SH, insomnia, irritability, decreased appetite.	Palma *et al* (118)
Droxidopa	Synthetic norepinephrine precursor.	Initially, 100 mg 3 times daily (at least 3 h before bedtime); titrate in increments of 100 mg 3 times daily every 24. Maximum dose of 600 mg 3 times daily.	SH, headache, dizziness, nausea, and fatigue; caution in heart failure and chronic renal failure.	Hauser *et al* (345)
Fludrocortisone	Synthetic mineralocorticoid. Volume expander that increases sodium and water reabsorption.	0.05-0.2 mg/day; minimal benefit observed with dosages beyond 0.2 mg/day.	SH, hypokalemia, renal failure, and edema; caution in congestive heart failure.	Lahrmann *et al* (32)
Midodrine	Direct α1-adrenergic receptor agonist.	2.5-15 mg twice or three times/day (dosed morning, midday, and 4 h before bedtime).	SH, piloerection (‘goose bumps’), scalp itching, and urinary retention; caution in congestive heart failure and chronic renal failure.	Brignole *et al* (346)
Pyridostigmine	Acetyl-cholinesterase inhibitor. Marginal efficacy in nOH.	30-60 mg twice or three times/day.	Abdominal cramps, diarrhea, sialorrhea, excessive sweating, urinary incontinence.	Singer *et al* (313)
B, Neurogenic SH				
Drug	Mechanism	Recommendations	Adverse effects	Authors/(Refs.)
Captopril	Angiotensin-converting enzyme inhibitor.	25 mg at bedtime.	Coughing resolves after discontinuing the drug; low BP; flushing (sudden warmth, redness, or tingly feeling); low blood cell counts; and decreased sense of taste.	Gibbons *et al* (262)
Eplerenone	Mineralocorticoid receptor antagonist.	50 mg at bedtime.	Dizziness. Diarrhea. Coughing. Flu-like symptoms, such as fever and body aches. Tiredness.	Arnold *et al* (321)
Hydralazine	Vasodilator. Peripheral smooth-muscle relaxant.	10-25 mg at bedtime.	Headache Nausea. Vomiting. Diarrhea. Loss of appetite.	Shannon *et al* (347)
Losartan	Angiotensin II receptor antagonist, acting on AT1 receptor subtype.	25-50 mg at bedtime.	Back pain. Dizziness. Cough. Acute kidney injury. Nasal congestion. Fatigue. Stuffy nose. Hyperkalemia.	Arnold *et al* (348)
Nifedipine	Calcium channel blocker.	30 mg at bedtime.	Angioedema. Flushing. Gingival hyperplasia. Syncope.	Jordan *et al* (349)

nOH, neuxrogenic orthostatic hypotension; SH, supine hypertension.

**Table V:** Summary of pharmacological treatment of cognitive impairment in PD.

Drug	Mechanism	FDA	Recommendations	Adverse effects	Authors/(Refs.)
Donepezil	AChEI.	FDA-approved for Alzheimer's disease but not PDD. Reasonable to consider in PDD.	Initial: 5 mg once daily; may increase to 10 mg once daily after 4 to 6 weeks.	Nausea, vomiting, and tremor. Rare bradycardia.	Dubois *et al* (350)
Galantamine	AChEI.	FDA-approved for Alzheimer's disease but not PDD. Reasonable to consider in PDD.	Initial: 4 mg twice daily for 4 weeks; if tolerated, increase to 8 mg twice daily for ≥4 weeks; if tolerated, increase to 12 mg twice daily. Range: 16 to 24 mg daily in 2 divided doses.	Nausea, vomiting, and tremor. Rare bradycardia.	Wang *et al* (351)
Rivastigmine	AChEI.	Only FDA-approved medication for cognitive symptoms of PDD.	Initial: 1.5 mg twice daily; may increase by 3 mg daily (1.5 mg per dose) every 4 weeks based on tolerability (maximum recommended dose: 6 mg twice daily).	Nausea, vomiting, and tremor. Rare bradycardia.	Litvan *et al* (352)
Memantine	N-Methyl-D-aspartate (NMDA) receptor antagonist.	FDA approved for moderate to severe Alzheimer's disease but not for PDD.	Initial: 5 mg once daily; increase dose by 5 mg weekly as tolerated to a target maximum dose of 20 mg/day.	Widely prescribed partly due to tolerability. Occasional worsening of hallucinations reported.	Emre *et al* (353)

AChEI, acetylcholinesterase inhibitor; FDA, Food and Drug Administration; PDD, Parkinson's disease dementia.

## Data Availability

Not applicable.
